# *POLE* proofreading mutation, immune response and prognosis in endometrial cancer

**DOI:** 10.1080/2162402X.2015.1072675

**Published:** 2015-08-12

**Authors:** Inge C. van Gool, Tjalling Bosse, David N. Church

**Affiliations:** a Department of Pathology, Leiden University Medical Center, Leiden, the Netherlands; b Molecular and Population Genetics Laboratory, The Wellcome Trust Centre for Human Genetics, University of Oxford, Oxford, UK

**Keywords:** DNA polymerase ε, *POLE*, ultramutation, neopeptide

## Abstract

Endometrial cancers (ECs) with *POLE* proofreading mutations are typified by ultramutation and excellent prognosis. We investigated whether these were related, and found that *POLE*-mutant ECs display a robust T cell response that corresponds to an enrichment of antigenic tumor neopeptides. Enhanced immunogenicity may explain the favorable outcome of *POLE*-mutant ECs.

The proofreading exonuclease activity intrinsic to the replicative DNA polymerases epsilon and delta (Pols ε and δ) is essential to maintain fidelity of DNA replication and prevent mutagenesis. While a role for defective polymerase proofreading in human cancer has long been postulated, this has only recently been confirmed, with the demonstration that germline mutations in the exonuclease domains of *POLE* and *POLD1* (which encode the principal subunits of Pols ε and δ respectively) predispose to cancer.^1^ Subsequently, we and others have shown that somatic *POLE* proofreading mutations occur in 7–12% ECs,^2,3^ 1–2% colorectal cancers (CRCs), as well as cancers of the brain, stomach and pancreas (TCGA unpublished, http://www.cbioportal.org, accessed June 2015). In keeping with the essential contribution of polymerase proofreading to replication fidelity, *POLE* proofreading-mutant ECs are ultramutated.^3^ However, perhaps less predictably, they also have an excellent prognosis.^3,4^ We hypothesized that these two characteristics may be related—more specifically, that tumor neopeptides caused by ultramutation may stimulate a cytolytic immune response, analogous to previous observations in hypermutated mismatch repair-deficient CRCs.^5^ In a recent study,^6^ we investigated this in two large EC cohorts.

Following the observation that *POLE* proofreading-mutants had a higher density of tumor-infiltrating lymphocytes (TILs) than other ECs, we confirmed that this represented a CD8^+^ cytotoxic T cell infiltrate likely to be capable of cytolytic activity, as evidenced by co-staining for the activation marker TIA-1. Consistent with these data, examination of RNAseq data from the independent TCGA EC series confirmed significant enrichment for immune-related pathways and a highly specific 200-gene tumor T cell infiltration signature in *POLE* proofreading-mutant ECs. This analysis also demonstrated that *POLE*-mutant tumors displayed significantly increased expression of CD8A (gene) and other T cell cytotoxic differentiation and effector markers known to predict favorable outcome in cancer,^7^ including T-bet, Eomes, IFNγ, perforin and granzymes B, H, K and M. Using a bio-informatic approach to investigate the possible contribution of antigenic tumor neopeptides to the antitumor immune response, we found that *POLE* proofreading-mutant ECs were predicted to display substantially more antigenic peptides than other ECs, providing a potential explanation for our findings.

Taken together, our data suggest that enhanced immunogenicity contributes to the excellent prognosis of *POLE* proofreading-mutant ECs, and are concordant with a recent study, which showed that dendritic cells pulsed by *POLE*-mutant tumor lysates stimulated greater CD4^+^ and CD8^+^ cell proliferation than those pulsed by ECs lacking *POLE* mutations.^8^ However, this begs the question of why *POLE-*mutant ECs are not eliminated by this enhanced cytotoxic T cell response? We found no evidence of an increased frequency of loss of HLA class I protein expression in *POLE*-mutant ECs, and functional mutations in the antigen presentation machinery also appeared relatively uncommon (2 of 18 cases). In contrast, we found striking increases in the expression of immunosuppressive checkpoint molecules and Treg markers, including LAG3, TIM-3, TIGIT, PD1, CTLA4 and FOXP3, in *POLE*-mutant ECs, suggesting that this may be the principal mechanism of immune evasion in these tumors.

In short, our data suggest that *POLE* proofreading-mutant ECs evoke a striking antitumor immune response, which is likely to contribute at least partly to their excellent prognosis (Fig. 1). In addition to validating our results in further independent EC series, it will be important to determine whether an enhanced cytotoxic T cell reaction also occurs in other *POLE* proofreading-mutant cancers. Interestingly, recent data suggest this may be the case in glioblastomas.^9^ Given the association between benefit from immune checkpoint inhibitors and tumor mutation burden,^10^ our study also suggests that the few patients with advanced or recurrent *POLE* proofreading-mutant cancers may be promising candidates for these agents. Finally, a pressing question is why some *POLE*-mutant tumors do not appear to stimulate as potent an immune reaction as others? Is it simply that these tumors are less mutated? Or do they harbor novel mechanisms of immune escape? Thus, while much remains unknown, further study of *POLE*-mutant cancers may provide insights into antitumor immune response and evasion that are generalizable more broadly, with potential benefits for a wide range of cancer patients.

## Figures and Tables

**Figure 1.**
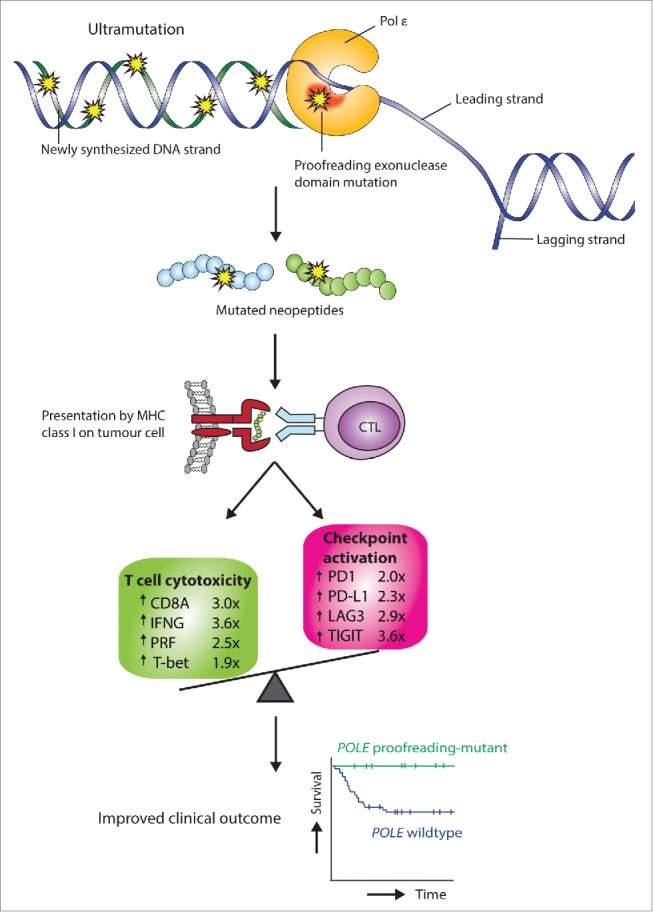
Possible mechanism linking *POLE* proofreading mutation, immune response and favorable endometrial cancer prognosis. *POLE* encodes the catalytic and proofreading subunit of DNA polymerase ε (Pol ε), the leading strand replicase in humans. Cancer-associated *POLE* exonuclease domain mutations perturb proofreading activity, resulting in tumor ultramutation. Enhanced presentation of mutated antigenic neopeptides stimulates both a cytolytic T cell response and upregulation of immunosuppressive checkpoints; however, increased effector cytokine expression (not shown) suggests that the T cell response is functional and at least partly contributes to the favorable prognosis of *POLE* proofreading-mutant endometrial cancers.
